# Genetic diversity and population structure of maize inbred lines using phenotypic traits and single nucleotide polymorphism (SNP) markers

**DOI:** 10.1038/s41598-023-44961-3

**Published:** 2023-10-19

**Authors:** Sweetbird Phindile Dube, Julia Sibiya, Funso Kutu

**Affiliations:** 1 School of Agricultural, Earth and Environmental Sciences, University of KwaZulu-Natal, Pietermaritzburg, 3209 South Africa; 2 School of Agricultural Sciences, University of Mpumalanga, Nelspruit, 1200 South Africa

**Keywords:** Genetics, Molecular biology, Plant sciences

## Abstract

Understanding germplasm’s genetic diversity is essential for developing new and improved cultivars with stable yields under diverse environments. The objective of this study was to determine the genetic diversity and population structure of 128 maize inbred lines sourced from the International Institute of Tropical Agriculture (IITA), the International Maize and Wheat Improvement Centre (CIMMYT), and the University of KwaZulu-Natal (UKZN) using 11,450 informative single nucleotide polymorphism (SNP) markers. The inbred lines revealed highly significant (p < 0.001) levels of variability for the key phenotypic traits. The SNP markers had a mean gene diversity (GD) and polymorphic information content (PIC) of 0.40 and 0.31, respectively, indicating the existence of substantial genetic variation across the germplasm panel. The model-based population structure analysis identified three subpopulations (K = 3) among the inbred lines. This corroborated the phylogenetic analysis using phenotypic traits and molecular markers which classified the inbred lines into three groups. The findings of this study identified considerable genetic diversity for the selection of inbred lines with favourable alleles for multiple traits and could be useful to initiate marker-assisted selection (MAS) to identify significant loci associated with agronomic performance and multiple-stress tolerance.

## Introduction

In South Africa, the maize (*Zea mays* L.) crop is a significant employer and source of foreign currency due to its multiplier effects. The crop has strong linkages throughout the economy, upstream to the input industries and downstream into the milling, animal feed, and food processing industries^1, 2^. The hectarage planted with maize in South Africa varies depending on weather and market conditions, but on average, 2.5 to 2.75 million hectares of hybrid maize are planted each year, yielding 12 million tons of grain^3^. This translates to surplus maize exported to Zimbabwe, Botswana, Lesotho, Namibia, Eswatini, and Mozambique. South Africa is the only country in Africa that is in the top 10 of maize producers in the world^4^. However, climate change-induced biotic and abiotic stresses threaten maize productivity in tropical areas. Maize is severely affected by extreme weather events such as high temperatures, and unpredictable rainfall patterns, resulting in heat and drought stress or flooding^5^. Approximately 90% of maize in South Africa is produced on dry land under rain-fed conditions^6^.

Rainfall has decreased drastically over the past few years causing a dramatic decline in maize volumes due to the El Nino induced droughts. The variations in the frequency and amount of rainfall received have deleterious effects on maize grain yields mainly when water deficits occur during flowering (anthesis and silking)^7^. In addition, frequent droughts and the rise in atmospheric temperatures create conducive conditions for spawning of pests, diseases, and parasitic weeds causing severe maize grain yield losses^8^. Maize ear rots and stalk rots are pathogens showing an increasing impact in changing climates^9^. In Southern Africa, due to changes in environmental conditions, Refs.^10, 11^ observed an increase in the incidence and severity of maize foliar diseases such as Phaeosphaeria leaf spot (PLS; causal agent *Phaeosphaeria maydis* (Henn.) Rane, Payak & Renfro) and northern leaf blight (NLB; causal agent *Exserohilum turcicum* Pass. Leornard & Snuggs) with previously resistant cultivars being affected. Thus, the selection of germplasm with improved resistance or tolerance to biotic and abiotic stresses is critical in developing resilient maize production systems adapted to climate change-induced stresses. The sub-Saharan Africa is a centre of diversity for tropical and subtropical maize which has evolved stress adaptation genes for wide adaptation in the harsh savannah climates. Hence, this germplasm can expand the genetic diversity required for the development of climate-resilient maize cultivars in South Africa.

The Consultative Group of International Agricultural Research (CGIAR) centers comprising the International Maize and Wheat Improvement Centre (CIMMYT) and International Institute of Tropical Agriculture (IITA) have contributed immensely to the genetic improvement of tropical maize in Africa. Several inbred lines with tolerance to a diverse range of biotic and abiotic stresses have been developed by CIMMYT and IITA^12^. The CIMMYT program in Africa is divided into the East and Southern Africa regional hubs. Yield, drought, heat, low N, Low P, *Striga*, grey leafspot, maize streak virus, rusts, and ear rots have been the primary targets for selection at the two hubs^13^.

However, the recent fall armyworm outbreaks in Southern Africa and the maize lethal necrosis virus (MLN) in East Africa presented new challenges warranting immediate action. To date, several multiple-stress tolerant CIMMYT maize lines (CMLs), including CMLs 536–544 and 571–572, and 587–592, bred in Southern Africa are available for public and private maize breeding programs in Africa^14^. The MLN Screening facility in East Africa, Kenya identified notable resistance to the virus, and integrated the resistance genes into African-adapted germplasm. Currently, maize is a principal cereal in West Africa because of the Central Africa Maize Collaborative Research Network (WECAMAN) established by IITA and all the national programs in that region. The IITA’s breeding strategy of combining *Striga* resistance, earliness, drought- and low-N tolerance enhanced maize yield production in multiple stress environments. Maize grown in West and Central Africa should mature early before the onset of terminal drought which triggers *Striga* parasitic infections. It is noteworthy that both programs integrate elite exotic germplasm to expand the genetic and phenotypic elasticity of tropical and subtropical maize in the region. For instance, some of CIMMYT’s inbred lines developed in Africa are derived from Tuxpeño Sequía, and La Posta Sequía populations^14^, with the CIMMYT heterotic group A coming from populations and lines including the Tuxpeno, Kitale, BSSS (Iowa Stiff Stalk Synthetic), B73 and Salisbury white (N), in contrast the CIMMYT group B contains populations and lines from ETO, Ecuador 573, Lancaster, Mo17 and Southern cross (SC)^10, 15^. Elite exotic germplasm introduces valuable new alleles that broaden the genetic divergence required to exploit heterosis for hybrid breeding^16^.

South Africa is the top maize producer in SSA, due to a vibrant private sector-owned maize seed industry, bolstered by biotechnology. About 85% of the maize grown in South Africa is genetically modified (GM) maize^17^, containing GM traits such as glyphosate and insect resistance, thus improving grain yield. While these traits have resulted in considerable yield gains compared to conventional maize production, introgression of these traits will potentially result in deleterious genetic ‘bottlenecks’. In addition, most commercial breeding programs developed new inbred lines by recycling elite lines via pedigree which also narrows genetic divergence. Furthermore, most maize hybrid breeding programs, particularly in southern and eastern Africa, have utilized elite maize inbred lines from nine heterotic groups^10, 15^ and derived from the “P” heterotic group (derivatives from Natal Potchefstroom Pearl, the SC, N and K64r derivatives as well as the broader CIMMYT A and B groups^15, 18^ among others. There is a need, to broaden the genetic diversity of local germplasm by integrating tropical and subtropical germplasm adapted to SSA environments^19^. This is because native traits will be critical in improving the resilience of maize production systems under threat from the negative effects of climate change.

Knowledge of genetic relationships between local maize germplasm, and tropical and sub-tropical maize inbred lines will guide parental selection and the devising of appropriate breeding designs. Genetic diversity analysis differentiates breeding populations and assists in classifying inbred lines into definite heterotic groups^20, 21^. Trait phenotyping can be used in conjunction with molecular makers in the designation of entries into heterotic groups, particularly for highly heritable traits^22^. While morphological and agronomic polymorphisms are highly amenable to the environment and developmental stage of the plant, molecular markers, on the other hand, are very stable. Single nucleotide polymorphisms (SNPs) are the predominant form of naturally occurring genetic variation and offer adequate variation to distinguish closely related individuals. As a result, they have become the preferred DNA marker of choice for diversity studies. Additionally, SNPs are useful for studying genetic variation due to their low cost per data point, lower genotyping error rates, locus-specificity, high genomic abundance, potential for high throughput analysis, and codominance^23–25^.

Thus, SNPs provide an opportunity for the assessment of genetic diversity among local South African inbred lines and exotic tropical and subtropical maize germplasm developed for national maize breeding programs in SSA^26^. Gaining insight into how locally bred lines are related to lines developed by the CGIAR, suffices in the exploitation of heterosis in local breeding programs. Therefore, the objectives of this study were to assess genetic diversity and interrelationships present among locally bred maize inbred lines and the CGIAR tropical and sub-tropical maize lines adapted to SSA conditions, to determine heterotic groups and select unique genotypes for biotic and abiotic stress tolerance breeding. The set of local South African germplasm used in the study represents important genetic resources for use in public and private maize breeding programs in the country.

## Materials and methods

### Source of plant material

One hundred and twenty-eight (128) diverse maize inbred lines obtained from the International Maize and Wheat Improvement Centre (42), the International Institute of Tropical Agriculture (50), and the University of KwaZulu-Natal (36) were used in this study. The CIMMYT and IITA inbred lines were selected for their nutritional content and resistance/tolerance to biotic and/or abiotic stresses. The UKZN inbred lines were chosen to represent a sample of locally developed inbred lines to be used in commercial hybrid maize breeding. The CIMMYT lines were developed in Kenya and Zimbabwe (East and Southern Africa regional hubs, respectively) and were derived from the Tuxpeño Sequía, La Posta Sequía, and the Drought-Tolerant Population (DTP) yellow and white gene pools. The IITA germplasm was bred in Nigeria from several broad-based germplasm sources with resistance to *Striga* and MSV, as well as tolerance to drought, including TZE-W Pop DT STR C0, TZE-Y Pop DT STR C0, and TZE Comp 5-Y C6. The description of the germplasm used in the study is summarised in Table S1.

### Description of experimental sites phenotyping, data collection, and data analysis

The test genotypes were evaluated under field conditions to complement molecular maker data. Two field trials were established at Ukulinga Research farm (29° 40′ S, 30° 24′ E, 800 m) of UKZN and Cedara Research Station (29° 32′ S, 30° 17′ E, 1076 m) during the 2020/2021 cropping seasons. The experiments were arranged in 8 × 16 alpha lattice design with 2 replicates. Each plot consisted of 1 row, 5 m long, with inter and intra-row spacing of 0.8 and 0.3 m, respectively. The plant population density was 41,666.6 plants ha^−1^. Field management followed the recommended maize agricultural practices.

Plant count (PC) was recorded as the total number of plants per plot counted after thinning. Days-to-anthesis (DA) and days-to-silking (DS) were recorded as the number of days from emergence to the date when 50% of the plants in a plot had produced pollen and silks, respectively. Anthesis-silking interval (ASI) was calculated as days to silking minus days to anthesis. Plant height (PH) expressed in centimetres (cm) was measured from base to tassel branching to the soil surface to the top of the tassel of 10 representative plants selected from each plot. Scoring the severity of rust (RST) disease of maize was done using a scale of 1–9, where 1 = 0% of leaf surface diseased (no rust) and 9 = 81–100% of leaf surface diseased. Field weight (FW) was measured as the total weight of unshelled cobs in kilograms (kg). Cob count (CC) was counted as number of cobs per plot at harvest. The number of kernel rows per ear (KR) and kernels per row per ear (KRE) were determined as counts. Ear length (EL) was recorded (cm) as the length of a cob from the tip to the base using the ruler, while the diameter of the cob (ED) was measured using a vernier calliper (mm). Grain moisture (MOI) content was measured using a moisture tester during shelling. Shelling percentage (SHP) was calculated as field weight minus grain weight and the difference was expressed as a percentage. A sample of 100 kernels, grain weight per ear (GWE) from each plot was weighed. Grain yield (GY) expressed in t ha^−1^ was obtained from grain weight per plot adjusted to 12.5% grain moisture following CIMMYT (1999).

$$\mathrm{GY}=\left(\frac{\mathrm{GW}}{\mathrm{NP}}\right)\times 10\times \left(\frac{100-\mathrm{MO}}{87.5}\right),$$

where GY—grain yield (kg/ha), GW—grain weight at harvest (kg/ha), MO—moisture content (%) of grains at harvest, 87.5—Standard dry matter of grain at 12.5% as required by maize grain market authorities in South Africa, NP—Net plot area (number of rows × intra-row spacing × number of stations × inter-row spacing).

Data generated were subjected to analysis of variance after testing for normality and homogeneity of variance using R software. Trait BLUPs were computed using DeltaGen (https://www.deltagen.agr.nz). Furthermore, the association among traits was deduced using Pearson correlation coefficients on the corrplot package in R software^27^.

### Genotyping

#### DNA extraction, genotyping, and quality control

Fresh leaf samples were collected from 3 weeks old seedlings within each genotype and was shipped to SEQART AFRICA (https://www.seqart.net) in Nairobi, Kenya for DNA extraction and genotyping by sequencing. The DArTseq protocol was used to genotype samples using a SNP chip covering the ten maize chromosomes. The initial 50,941 SNPs from the genotyping-by-sequencing (GBS) pipeline were filtered by imputation to remove SNPs with 20% missing data and 5% minor allele frequency (MAF) using the *snp*Ready package on R^28^. A total of 11,450 informative SNP markers were used after data imputation. Individual genotypes with > 20% missing data were removed.

### Genetic diversity and population structure analysis

The polymorphic information content (PIC), major allele frequency, the number of alleles, heterozygosity, and gene diversity were estimated using the R package “adegenet”^29^. Analysis of molecular variance (AMOVA) and genetic diversity was performed using GenAlex^30^ after grouping the inbred lines based on the source of collection. Population structure was determined using the STRUCTURE v2.3.4 software^31^. The burn-in period and Markov Chain Monte Carlo (MCMC) length were set at 10,000 iterations, and the model was run by varying the number of clusters (K) from 1 to 10 with 10 alterations for each K. The appropriate K value was estimated by implementing the Evanno method using the STRUCTURE Harvester program^32^. A joint analysis of phenotypic and genotypic data was conducted. A phenotypic distance matrix was generated based on Gower’s distance, while the genotypic distance matrix was generated using Jaccard’s coefficient on the R software^27^. The phenotypic, and genotypic matrices were used to generate hierarchical clusters using the package “cluster” in R software^27^.

## Results

### Analysis of phenotypic traits and relationships among traits

The combined analysis of variance for the 15 quantitative traits studied is shown in Table 1. Analysis of variance revealed highly significant differences (p < 0.001) among genotypes for all the studied traits. The environmental factor exhibited significant differences for all the evaluated traits except for the days to anthesis (DA), ear length (EL), ear diameter (ED), and kernels per row per ear (KRE). The replicates revealed significant differences for days to days to 50% anthesis and silking (DA and DS), plant height (PH), cob count (CC), field weight (FW), grain weight per ear (GWE), and grain yield (GY). The genotype × environment (GxE) interaction displayed significant differences (p < 0.01) among all the traits except for days to 50% anthesis and silking (DA and DS), gain moisture (MOI), and shelling percentage (SHP).

**Table 1:** Analysis of variance showing mean square values for the 15 morpho-agronomic traits for 128 maize genotypes.

Source	DF	PC	DA	DS	ASI	PH	EL	ED	KR	KRE	CC	FW	MOI	GWE	SHP	GY
Genotypes (G)	127	26.8***	83.7***	82.8***	3.8***	4755.0***	28.9***	173.9***	19.6***	161.3***	82.6***	3.9***	9.4***	0.0***	73.1***	7.1***
Environment (E)	1	23.6*	4.3	356.1***	438.8***	74,165.0***	11.9	2.1	38.6**	5.0	109.7*	37.1***	962.5***	0.0***	1507.8***	57.4***
Rep (R)	1	12.5	143.4**	127.0**	0.5	18,296.0***	0.6	81.3	1.2	7.1	87.0*	6.2***	4.6	0.0**	0.4	11.4***
GxE	127	8.1*	19.9	21.3	2.1*	660.0***	7.1**	65.7*	6.8**	34.2	26.5***	0.6***	3.5	0.0*	15.7	1.5***
ExR	1	0.1	43.4	18.4	5.3	63,401.0***	3.4	196.1*	26.7*	88.6	32.5	1.2	38.9**	0.0**	4.9	2.0
REP:BLK	42	6.4	26.8*	21.7	2.2*	623.0**	7.3*	71.3*	5.5	39.7	29.4**	0.5*	3.8	0.0*	15.1	1.5**
Residuals	212	5.8	18.0	16.8	1.5	323.0	4.8	48.8	4.3	28.0	16.3	0.3	4.1	0.0	14.2	0.9

Significant codes: 0 "***", 0.001 "**" and 0.01 "*".

The mean performance of the genotypes showed that days to 50% anthesis and silking (DA and DS) ranged from approximately 83 to 91 days. Anthesis and silking interval range from − 0.4 to 1.8 with a mean of 0.3. Plant height ranged from 99 to 274 cm with a mean of 179 cm. Cob count exhibited a mean of 15 and ranged between 3 and 27 per plot. Grain weight per cob ranged from 0 to 0.2 g with a mean of 0.1 g. Considerable variation was observed for grain yield ranging from 0.9 to 5.9 t ha^−1^ with a mean performance of 2.8 t ha^−1^. The top performing genotypes in terms of grain yield comprised TZISTR1190, TZISTR1261, CML540, CML571, and TZISTR1119, recording 5.9, 5.8, 5.6, 5.6, and 5.5 t ha^−1^, respectively. The least performing genotypes were CML545, 18 UK1-54 and TZISTR1162 recording 1.3, 0.9 and 0.9 t ha^−1^ respectively. The mean performances for all the phenotypic traits of the genotypes are summarized in Table S2.

**Figure 1:**
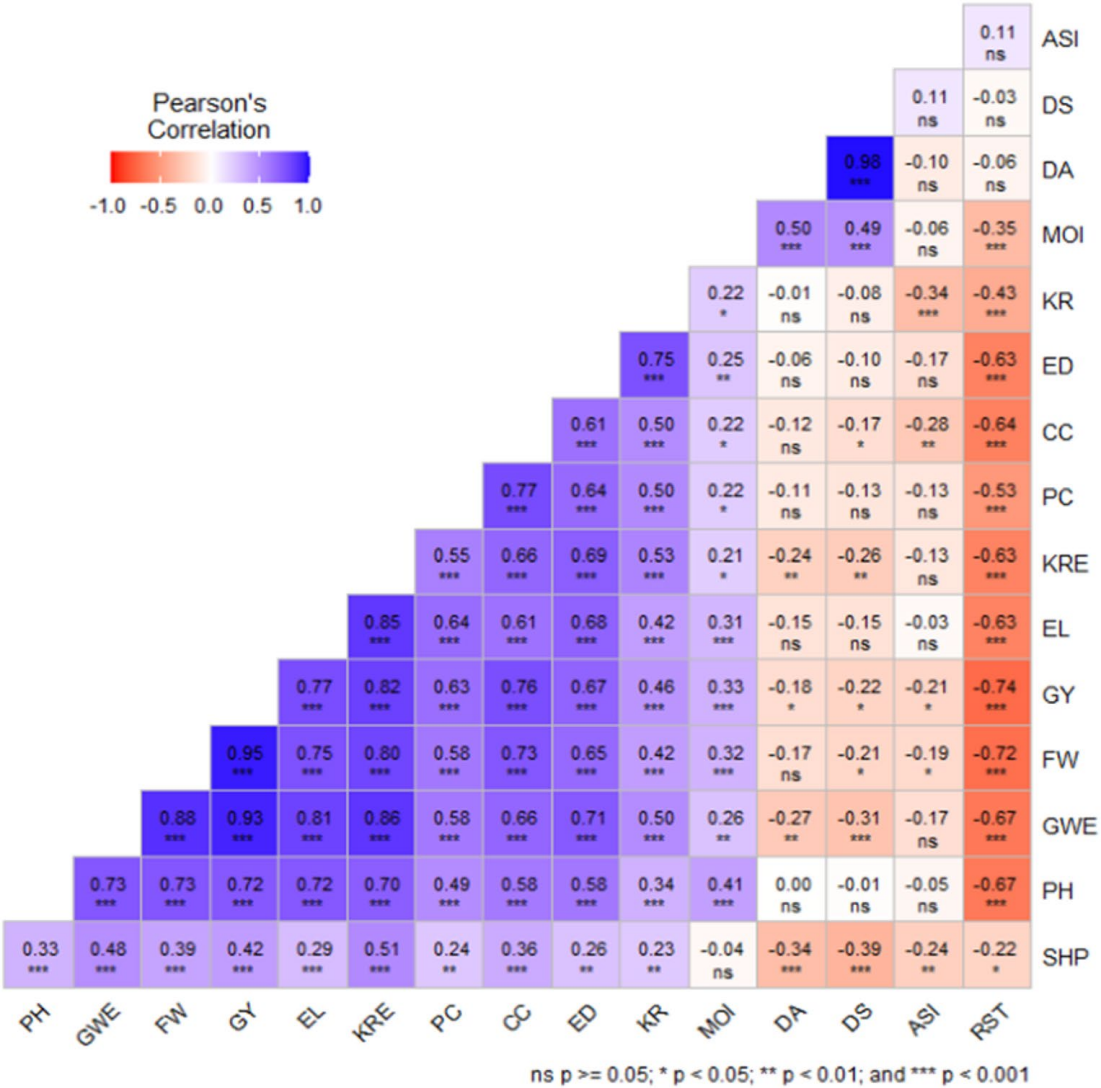
Heat map showing positive (blue squares) and negative (red squares) correlations.

The association among the 16 measured phenotypic traits is depicted by the heat map of Pearson correlation coefficients. The blue and red squares are negative and positive correlations, respectively (Fig. 1). Strong correlations were observed between grain yield and cob count (0.76), plant height (0.72), ear length (0.77), ear diameter (0.67), and field weight (0.95). However, grain yield was negatively and significantly correlated with days to anthesis (− 0.18), days to silking (− 0.22), anthesis-silking interval (− 0.21) and rust (− 0.74).

### Genetic diversity analysis

#### Population structure and genetic diversity analyses

The gene diversity (GD) ranged between 0.03 and 0.50, with a mean of 0.40. While the polymorphic information content (PIC) range was between 0.03 and 0.38, with a mean of 0.31. The minor allele frequency (MAF) ranged from 0.01 to 0.50, with an average value of 0.34. The observed heterozygosity had the mean average of 0.67 varied between 0.59 and 0.83. Fixation index (F) ranged from − 1.1 to − 0.5 with a mean of − 0.69 (Table 2). The results from AMOVA displayed significant genetic differences among individuals and populations (Table 3). Two percent of the variance was due to genetic differentiation among the populations, in contrast individuals within the panel accounted for 98% of the variance. The pairwise population matrix of Nei Genetic Distance was the highest between genotypes from CIMMYT and UKZN (0.05) followed by IITA and UKZN (0.04) and lastly between CIMMTY and IITA with 0.03. The greatest genetic identity was observed between CIMMYT and IITA (0.97) followed by IITA and UKZN (0.96) and the least was observed between CIMMTY and UKZN (0.95) (Table 4).

**Table 2:** Diversity indices statistics of the maize inbred lines based on SNP markers, *GD* gene diversity, *PIC* polymorphic information content, *MAF* minor allele frequency, *Ho* observed heterozygosity,* F* Fixation index.

Statistics	GD	PIC	MAF	Ho	F
Mean	0.40	0.31	0.34	0.67	− 0.69
Lower	0.03	0.03	0.01	0.59	− 1.1
Higher	0.50	0.38	0.50	0.83	− 0.5

**Table 3:** Molecular analysis of variance of maize populations based on 11,450 SNP markers.

Source	df	SS	MS	Est. var.	%
Among population	2	8460.69	4230.35	31.48	2
Within individuals	127	316,160.00	1638.13	1638.13	98
Total	253	421,428.05		1669.61	100

**Table 4:** Pairwise population matrix of nei genetic distance (lower diagonal) and genetic identity (above diagonal).

	UKZN	IITA	CIMMYT
UKZN	1	0.04	0.05
IITA	0.96	1	0.03
CIMMYT	0.95	0.97	1

**Figure 2:**
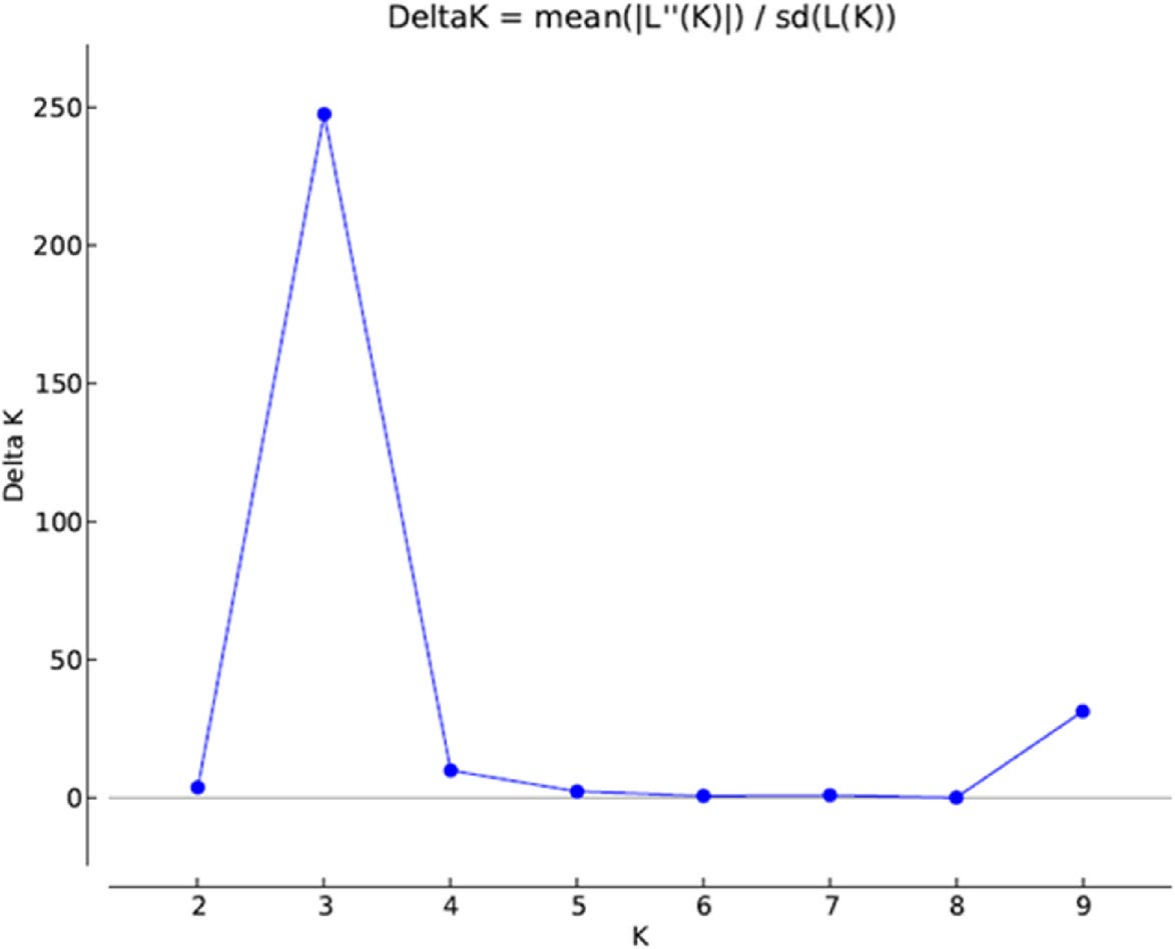
Delta K showing the number of populations.

**Figure 3:**
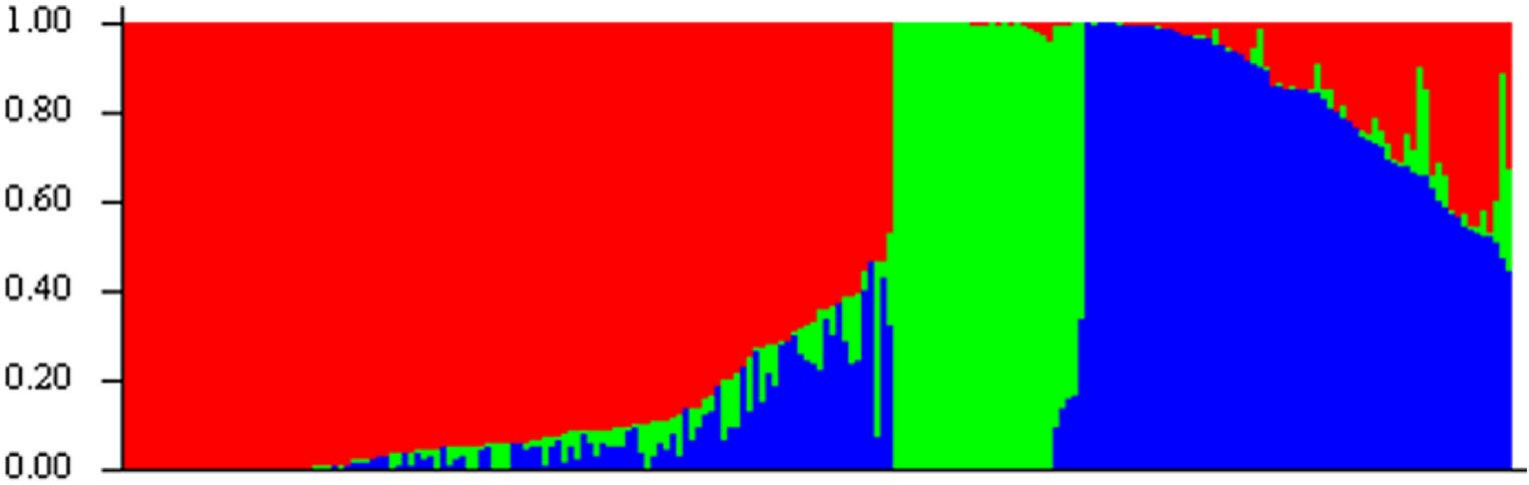
Bar plot of population sorted by kinship matrix.

The population structure analysis identified three subpopulations among the inbred lines based on the optimal K = 3 determined according to Evanno’s method (Figs. 2, 3). The distribution of genotypes into clusters was based on 70% kinship. Sub-populations 1, 2, and 3 comprised 54%, 16%, and 30% of the total genotypes, respectively. The allele frequency divergence between subpopulations and the expected heterozygosity between genotypes within the same subpopulations is presented in (Table 5). Subpopulations 1 and 2 exhibited the highest allele frequency divergence of 0.08, followed by subpopulations 1 and 3 with 0.07. The least allele frequency was recorded for subpopulations 2 and 3 with 0.12. Within the three subpopulations, the expected heterozygosity between genotypes varied from 0.24, 0.12, and 0.16 for subpopulations 1, 2 and 3, respectively (Table 5).

**Table 5:** Allele frequency divergence among sub-populations and expected heterozygosity (average distance) between genotypes within the same subpopulations.

	FST allele frequency divergence among subpopulations			Membership%
	I	II	III	
I	–	0.0810	0.0737	0.54
II	0.0810	–	0.1159	0.16
III	0.0737	0.1159		0.30

Expected heterozygosity within subpopulations				
	I	II	III	
	0.244	0.115	0.158	

### Cluster analysis of phenotypic and genotypic data for the 128 genotypes

**Figure 4:**
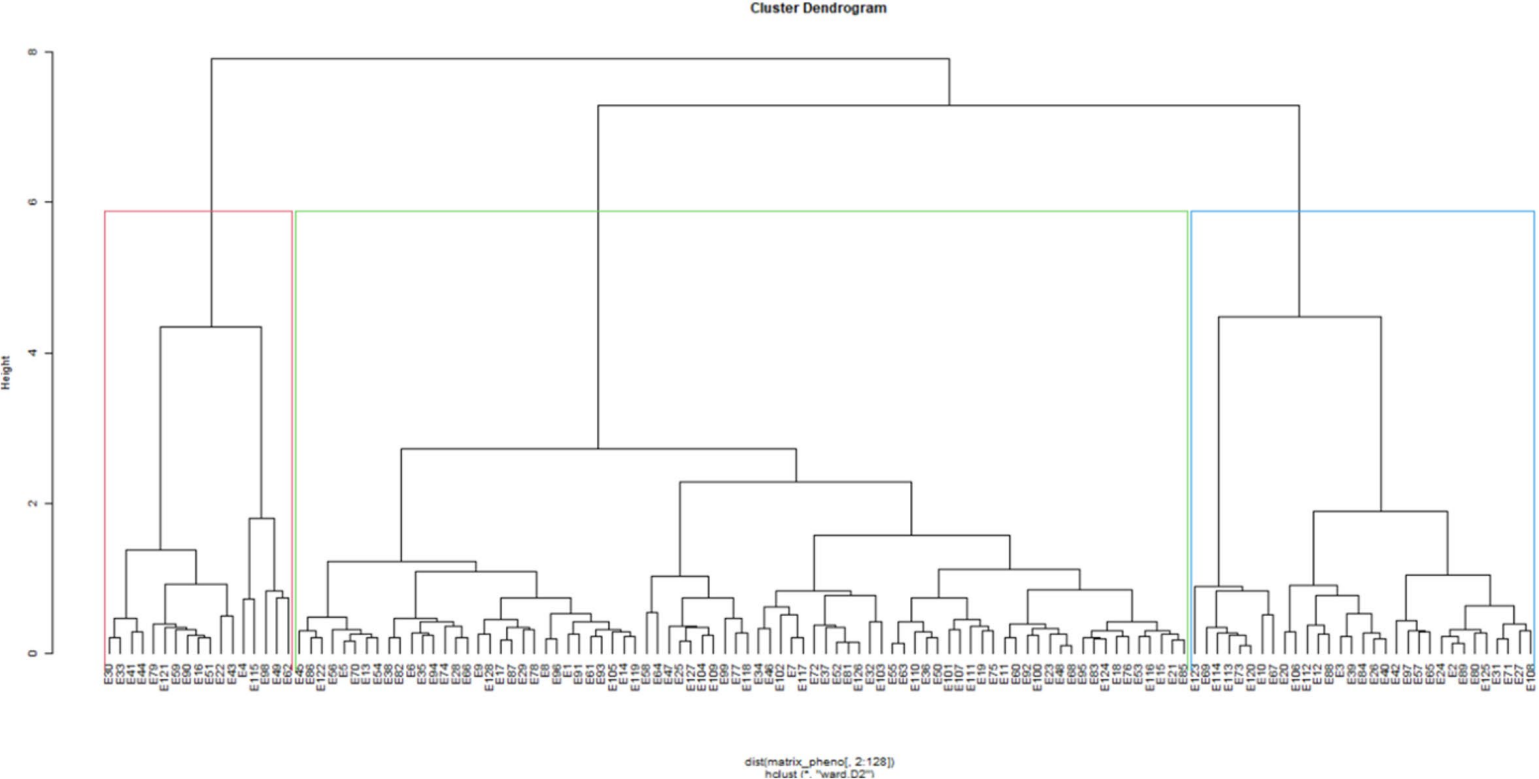
Dendrogram displaying the relationship between the 128 genotypes of maize based on the phenotypic matrix.

The dendrogram based on phenotypic data showed three clusters: I, II, and III, each consisting of 81%, 9%, and 10% membership, respectively (Fig. 4, Table S3). Cluster I was an admixture group with 103 genotypes, having 35 IITA, 36 CIMMYT, and 32 UKZN material. Most of the genotypes (TZSTRI102, 17CED MAK1-61/62, and 18 UK1-3) in this cluster were associated with high anthesis-silking interval (ASI) coupled with reduced grain yield. Cluster II consisted of most high yielding genotypes (TZISTR1261, TZISTR1261, CML540, CML571, and TZISTR1119), with 8 from IITA and 4 from CIMMYT. The third cluster comprised 13 genotypes associated with the lowest yield, the least being CML545 and 18 UK1-54 with each recording 0.9 t ha^−1^.

**Figure 5:**
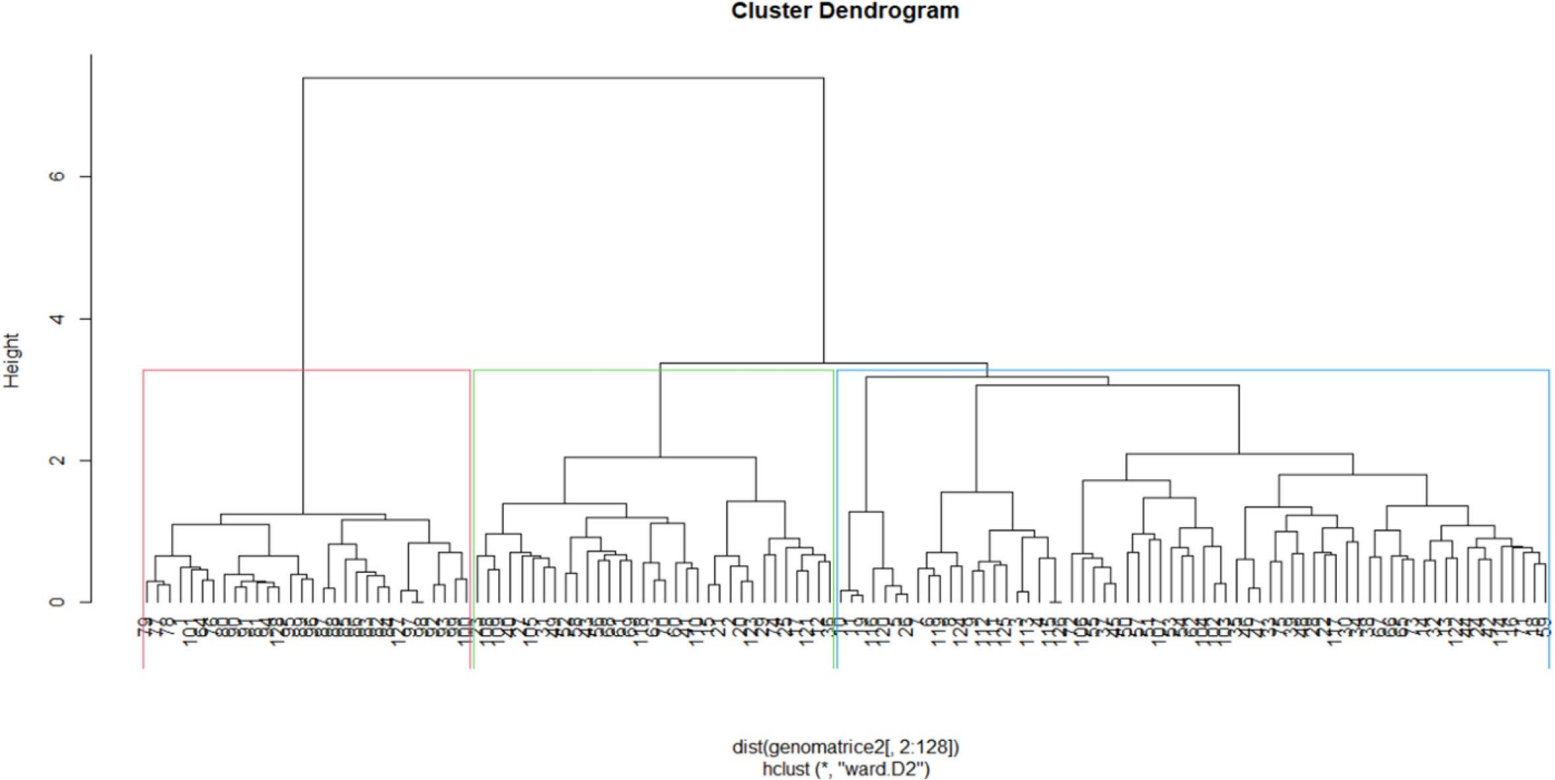
Dendrogram illustrating the relationship between the 128 genotypes of maize based on the genotypic matrix.

Based on the genetic data, the dendrogram showed that Clusters I, II, and III consisted of 23%, 26%, and 51% membership, respectively (Fig. 5, Table S4). The first cluster consisted of the 30 genotypes with 29 from UKZN and 1 from CIMMYT. The second cluster consisted of 14 and 19 genotypes from IITA and CIMMYT, respectively. The last cluster was the largest, with 65 genotypes, of which 35 were from IITA. The 25 and 5 genotypes were from CIMMYT and UKZN, respectively. The CIMMYT and IITA exchange a lot of planting material, which could be why they are grouped in the same cluster.

## Discussion

Genetic diversity studies are critical in identifying the individual genotypes among closely and distantly related groups for the start of new breeding initiatives. To breed novel hybrids with outstanding agronomic performance, multiple stress tolerance and high-quality yield, there is a need to understand the genetic interrelationships among the inbred lines. Hence, the rational of this study was to gain genetic relationships among South African-bred maize inbred lines, tropical and subtropical inbred lines developed across the African continent.

The study revealed significant genetic variation among the inbred lines using phenotypic traits and SNP markers. Analysis of variance showed highly significant differences among genotypes for grain yield and its associated component traits, indicating substantial genetic variation among the genotypes. Grain yield and its associated yield components are quantitatively inherited and are of polygenic inheritance which can enable selection between genotypes within a population^33^. The significant differences observed in days to 50% anthesis and silking (DA and DS) among genotypes are essential for crop development in drought-prone environments. Early flowering and maturity are desired characteristics because they contribute to drought escape. This is particularly important in the SSA context, adversely affected by climate change resulting in poor rainfall^34, 35^.

The phenotypic diversity observed in the test population was supported by the analysis of molecular variance. Genomic data is utilized in determining phylogenetic relationships in germplasm collections^36^. The high number of SNP markers utilized in this study enabled a more precise estimate of the population structure. Gene diversity ranged between 0.03 and 0.5, with a mean of 0.4. Reference^37^ reported similar findings with the genetic diversity varying from 0.01 to 0.5. The polymorphism information content (PIC) predicts the relevance of a genetic marker for linkage analysis^38, 39^. In this current study, the PIC average value was 0.31, indicating that the SNP markers employed could effectively discriminate between the genotypes. These results are in support of those reported by Refs.^39, 40^. Single nucleotide polymorphisms are bi-allelic; therefore, their PIC values are often lower than SSRs; however, they offer higher genetic resolution^41, 42^. The mean observed heterozygosity (Ho) of 0.67 reported in this study was considerably higher than those reported for inbred lines by Refs.^43, 44^. This suggest(s) that some of the inbred lines were not homozygous. Further selfing and substantial selection is strongly advised to fix these inbred lines^45^. The inbreeding coefficient (F) quantifies the likelihood that two alleles at every locus within an organism are identical by descent from the two parents’ shared ancestor(s)^46^. In this study, the F value ranged from − 1.1 to − 0.5, with an average of − 0.69. Reference^47^ similarly observed a negative F mean value of − 0.29. A negative F value in a population sample indicates the presence of excess heterozygotes^48^.

The panel of selected SNPs used in this study effectively revealed the polymorphisms existing among and within the inbred line populations. The within inbred line variation accounted for the most significant proportion of the variation observed compared to among population differences. A constant germplasm exchange between IITA and CIMMYT which may explain the lack of high genetic identity and low distance genetic divergence observed between the two centres. For example, parental germplasm used to initiate the CIMMYT program for drought and low soil N stress tolerance in Kenya was sourced from the CIMMYT Southern Africa regional centre in Zimbabwe and early maturing populations from the International Institute of Tropical Agriculture (IITA)^49^. Likewise, CIMMYT has been the major source of white maize germplasm for most SSA commercial seed companies because of the prominence of white maize in the region, particularly in Southern Africa^10, 15^. This presents genetic bottlenecks that limit breeding gains. Reference^50^ reported a wide divergence between Southern Africa sub-tropical germplasm and temperate maize lines suggesting the potential utility of temperate germplasm in expanding the genetic base of African germplasm. The combination of stress tolerance and high-yield performance can produce broad adaptation in hybrids for South Africa. The Water Efficient Maize for Africa (WEMA) program is a good example of the efficacy of synergy between the private and public sector germplasm. Under the project, Monsanto, and CIMMYT developed germplasm combining drought tolerance and optimum yield performance. The resultant hybrids have a 20% yield advantage under drought conditions and outperform most commercial hybrids in optimum environments^51^.

The SNP analysis revealed the existence of three subpopulations (K = 3). These results are in agreement with those reported by Ref.^37^ who reported the existence of 3 subpopulation among 94 early maturing tropical maize inbred lines using SNP markers. Similarly, the dendrogram grouped the genotypes into three primary clusters showing partial existence of origin as a source of diversity. In genetic diversity studies, reports of the grouping of genotypes according to their geographic origins are common^47^. However, the clustering of most inbred lines was not based on ancestry suggesting that maize inbred lines derived from the same populations do not always have the same selection. This also indicates that the study panel consisted of unique inbred lines. The UKZN inbred lines were bred for performance in high-potential environments while the CGIAR material was developed for adaptation in stress environments.

## Conclusions

The results of this study revealed that the maize inbred lines displayed phenotypic variation supported by genetic diversity, which will enable the selection and breeding of stress-tolerant maize hybrids. The genotypes exhibited highly significant variation in key agronomic traits such as DA, DS, PC, PH, and GY. High genetic distances between paired inbred lines demonstrated the distinctiveness of the evaluated genotypes and the availability of substantial genetic variation that could be utilized in the maize breeding program. The inbred lines were partially classified into three heterotic groups based on source background. Superior hybrids can be developed from inbred lines exhibiting the most comprehensive genetic distance within populations identified. Combining ability studies are recommended to confirm the agronomic performance and breeding values of the most divergent parental lines.

### Supplementary Information

Supplementary Tables.

## Data Availability

All relevant data are within the paper and its Supporting Information files. The use and collection of plant material comply with relevant institutional, national, and international guidelines and legislation.
