# Supplementary material for: Genetic diversity and population structure of maize inbred lines using phenotypic traits and single nucleotide polymorphism (SNP) markers
Source: Sci Rep. 2023 Oct 19;13:17851. doi: 10.1038/s41598-023-44961-3 (PMC10587089; doi:10.1038/s41598-023-44961-3)
Supplement: Supplementary file 1 — Supplementary Tables. [file 41598_2023_44961_MOESM1_ESM.docx]

**Appendix**

**Table S1: 128 maize inbred lines used in the study**

| **Entry** | **Genotypes** | **Source** | **Background** | **Entry** | **Genotypes** | **Source** | **Background** |
| --- | --- | --- | --- | --- | --- | --- | --- |
| 1 | 17CED MAK1-59/60 | UKZN | Yield and disease resistant | 65 | CML443 | CIMMTY | Provitamin-A and drought tolerant |
| 2 | TZISTR1263 | IITA | Striga resistant and drought tolerant | 66 | CML542 | CIMMTY | Provitamin-A and drought tolerant |
| 3 | TZISTR1157 | IITA | Striga resistant and drought tolerant | 67 | CML571 | CIMMTY | Provitamin-A and drought tolerant |
| 4 | TZISTR1162 | IITA | Striga resistant and drought tolerant | 68 | CML544 | CIMMTY | Provitamin-A and drought tolerant |
| 5 | TZISTR1175 | IITA | Striga resistant and drought tolerant | 69 | CML548 | CIMMTY | Provitamin-A and drought tolerant |
| 6 | TZISTR1177 | IITA | Striga resistant and drought tolerant | 70 | A1220-4CYL | CIMMTY | Provitamin-A and drought tolerant |
| 7 | TZISTR1174 | IITA | Striga resistant and drought tolerant | 71 | CML541 | CIMMTY | Provitamin-A and drought tolerant |
| 8 | TZISTR1163 | IITA | Striga resistant and drought tolerant | 72 | CML539 | CIMMTY | Provitamin-A and drought tolerant |
| 9 | TZISTR1166 | IITA | Striga resistant and drought tolerant | 73 | CML540 | CIMMTY | Provitamin-A and drought tolerant |
| 10 | TZISTR1190 | IITA | Striga resistant and drought tolerant | 74 | CML451 | CIMMTY | Provitamin-A and drought tolerant |
| 11 | TZISTR1199 | IITA | Striga resistant and drought tolerant | 75 | CML304 | CIMMTY | Provitamin-A and drought tolerant |
| 12 | TZISTR1231 | IITA | Striga resistant and drought tolerant | 76 | 18 UK1-1 | UKZN | Yield and disease resistant |
| 13 | TZISTR1232 | IITA | Striga resistant and drought tolerant | 77 | 18 UK1-3 | UKZN | Yield and disease resistant |
| 14 | TZISTR1244 | IITA | Striga resistant and drought tolerant | 78 | 18 UK1-5 | UKZN | Yield and disease resistant |
| 15 | TZISTR1262 | IITA | Striga resistant and drought tolerant | 79 | 18 UK1-6 | UKZN | Yield and disease resistant |
| 16 | TZISTR1223 | IITA | Striga resistant and drought tolerant | 80 | 18 UK1-8 | UKZN | Yield and disease resistant |
| 17 | TZSTRI109 | IITA | Striga resistant and drought tolerant | 81 | 18 UK1-9 | UKZN | Yield and disease resistant |
| 18 | TZSTRI110 | IITA | Striga resistant and drought tolerant | 82 | 18 UK1-14 | UKZN | Yield and disease resistant |
| 19 | TZSTRI111 | IITA | Striga resistant and drought tolerant | 83 | 18 UK1-15 | UKZN | Yield and disease resistant |
| 20 | TZSTRI112 | IITA | Striga resistant and drought tolerant | 84 | 18 UK1-16 | UKZN | Yield and disease resistant |
| 21 | TZSTRI114 | IITA | Striga resistant and drought tolerant | 85 | 18 UK1-17 | UKZN | Yield and disease resistant |
| 22 | TZSTRI117 | IITA | Striga resistant and drought tolerant | 86 | 18 UK1-18 | UKZN | Yield and disease resistant |
| 23 | TZISTR25 | IITA | Striga resistant and drought tolerant | 87 | 18 UK1-21 | UKZN | Yield and disease resistant |
| 24 | TZISTR1001 | IITA | Striga resistant and drought tolerant | 88 | 18 UK1-24 | UKZN | Yield and disease resistant |
| 25 | TZISTR1003 | IITA | Striga resistant and drought tolerant | 89 | 18 UK1-29 | UKZN | Yield and disease resistant |
| 26 | TZISTR1004 | IITA | Striga resistant and drought tolerant | 90 | 18 UK1-32 | UKZN | Yield and disease resistant |
| 27 | TZISTR1011 | IITA | Striga resistant and drought tolerant | 91 | 18 UK1-33 | UKZN | Yield and disease resistant |
| 28 | TZISTR1018 | IITA | Striga resistant and drought tolerant | 92 | 18 UK1-34 | UKZN | Yield and disease resistant |
| 29 | TZEEI21 | IITA | Striga resistant and drought tolerant | 93 | 18 UK1-35 | UKZN | Yield and disease resistant |
| 30 | TZEEI14 | IITA | Striga resistant and drought tolerant | 94 | 18 UK1-37 | UKZN | Yield and disease resistant |
| 31 | TZEEI34 | IITA | Striga resistant and drought tolerant | 95 | 18 UK1-42 | UKZN | Yield and disease resistant |
| 32 | TZEEI10 | IITA | Striga resistant and drought tolerant | 96 | 18 UK1-46 | UKZN | Yield and disease resistant |
| 33 | TZDEEI55 | IITA | Striga resistant and drought tolerant | 97 | 18 UK1-49 | UKZN | Yield and disease resistant |
| 34 | TZDEEI50 | IITA | Striga resistant and drought tolerant | 98 | 18 UK1-54 | UKZN | Yield and disease resistant |
| 35 | CLHP0302 | CIMMTY | Provitamin-A and drought tolerant | 99 | 18 UK1-55 | UKZN | Yield and disease resistant |
| 36 | CLHP0310 | CIMMTY | Provitamin-A and drought tolerant | 100 | 18 UK1-56 | UKZN | Yield and disease resistant |
| 37 | CLHP0003 | CIMMTY | Provitamin-A and drought tolerant | 101 | 18 UK1-57 | UKZN | Yield and disease resistant |
| 38 | CLHP00378 | CIMMTY | Provitamin-A and drought tolerant | 102 | 18 UK1-2-10 | UKZN | Yield and disease resistant |
| 39 | CLHP0156 | CIMMTY | Provitamin-A and drought tolerant | 103 | 18 UK1-2-12 | UKZN | Yield and disease resistant |
| 40 | CLHP0113 | CIMMTY | Provitamin-A and drought tolerant | 104 | 18 UK1-2-13 | UKZN | Yield and disease resistant |
| 41 | CLHP0364 | CIMMTY | Provitamin-A and drought tolerant | 105 | 18 UK3-2-2 | UKZN | Yield and disease resistant |
| 42 | CLHP0343 | CIMMTY | Provitamin-A and drought tolerant | 106 | CLHP0221 | UKZN | Yield and disease resistant |
| 43 | CLHP0049 | CIMMTY | Provitamin-A and drought tolerant | 107 | CLHP0312 | UKZN | Yield and disease resistant |
| 44 | CLHP0350 | CIMMTY | Provitamin-A and drought tolerant | 108 | CML486 | UKZN | Yield and disease resistant |
| 45 | CLHP0005 | CIMMTY | Provitamin-A and drought tolerant | 109 | CLHP0303 | CIMMTY | Provitamin-A and drought tolerant |
| 46 | CLHP0022 | CIMMTY | Provitamin-A and drought tolerant | 110 | HA04A-2107-36 | IITA | Provitamin-A and drought tolerant |
| 47 | CLHP0020 | CIMMTY | Provitamin-A and drought tolerant | 111 | TZISTR1160 | IITA | Striga resistant and drought tolerant |
| 48 | CLHP0058 | CIMMTY | Provitamin-A and drought tolerant | 112 | TZISTR1165 | IITA | Striga resistant and drought tolerant |
| 49 | CLHP0326 | CIMMTY | Provitamin-A and drought tolerant | 113 | TZISTR1261 | IITA | Striga resistant and drought tolerant |
| 50 | CML538 | CIMMTY | Provitamin-A and drought tolerant | 114 | TZISTR1119 | IITA | Striga resistant and drought tolerant |
| 51 | CML440 | CIMMTY | Provitamin-A and drought tolerant | 115 | TZSTRI104 | IITA | Striga resistant and drought tolerant |
| 52 | CML312 | CIMMTY | Provitamin-A and drought tolerant | 116 | TZSTRI108 | IITA | Striga resistant and drought tolerant |
| 53 | CML566 | CIMMTY | Provitamin-A and drought tolerant | 117 | TZSTRI113 | IITA | Striga resistant and drought tolerant |
| 54 | CML441 | CIMMTY | Provitamin-A and drought tolerant | 118 | TZSTRI115 | IITA | Striga resistant and drought tolerant |
| 55 | CML537 | CIMMTY | Provitamin-A and drought tolerant | 119 | TZISTR1154 | IITA | Striga resistant and drought tolerant |
| 56 | CML547 | CIMMTY | Provitamin-A and drought tolerant | 120 | TZISTR1161 | IITA | Striga resistant and drought tolerant |
| 57 | CML390 | CIMMTY | Provitamin-A and drought tolerant | 121 | TZISTR1224 | IITA | Striga resistant and drought tolerant |
| 58 | CML442 | CIMMTY | Provitamin-A and drought tolerant | 122 | TZISTR1248 | IITA | Striga resistant and drought tolerant |
| 59 | CML504 | CIMMTY | Provitamin-A and drought tolerant | 123 | TZISTR1275 | IITA | Striga resistant and drought tolerant |
| 60 | I-137 | CIMMTY | Provitamin-A and drought tolerant | 124 | TZISTR1164 | IITA | Striga resistant and drought tolerant |
| 61 | CML536 | CIMMTY | Provitamin-A and drought tolerant | 125 | TZISTR1159 | IITA | Striga resistant and drought tolerant |
| 62 | CML545 | CIMMTY | Provitamin-A and drought tolerant | 126 | TZSTRI102 | IITA | Striga resistant and drought tolerant |
| 63 | CML550 | CIMMTY | Provitamin-A and drought tolerant | 127 | 17CED MAK1-61/62 | UKZN | Yield and disease resistant |
| 64 | MAK1-122 | CIMMTY | Provitamin-A and drought tolerant | 128 | 17CED MAK1-48/47 | UKZN | Yield and disease resistant |

**Table S2: The BLUPs for all the phenotypic traits of the 128 genotypes studied**

| **Genotypes** | **PC** | **DA** | **DS** | **ASI** | **PH** | **EL** | **ED** | **KR** | **KRE** | **CC** | **FW** | **MOI** | **GWE** | **SHP** | **RST** | **GY** |
| --- | --- | --- | --- | --- | --- | --- | --- | --- | --- | --- | --- | --- | --- | --- | --- | --- |
| TZISTR1190 | 14,1 | 84,1 | 84,0 | 0,2 | 244,7 | 16,6 | 45,6 | 14,1 | 34,3 | 22,3 | 2,8 | 13,5 | 0,2 | 82,6 | 3,3 | 5,9 |
| TZISTR1261 | 14,8 | 88,7 | 89,1 | 0,4 | 242,1 | 17,4 | 45,2 | 14,6 | 33,7 | 16,5 | 3,1 | 14,9 | 0,2 | 81,7 | 4,0 | 5,8 |
| CML540 | 13,7 | 87,4 | 87,2 | 0,0 | 228,0 | 15,1 | 45,3 | 15,9 | 33,5 | 20,3 | 2,4 | 14,4 | 0,1 | 83,0 | 3,0 | 5,6 |
| CML571 | 14,3 | 90,3 | 90,0 | -0,1 | 228,0 | 16,6 | 41,3 | 16,0 | 34,7 | 26,8 | 2,1 | 15,7 | 0,1 | 83,1 | 3,5 | 5,6 |
| TZISTR1119 | 12,7 | 87,9 | 87,8 | 0,0 | 252,3 | 16,2 | 46,4 | 14,6 | 36,2 | 19,8 | 3,9 | 13,4 | 0,1 | 81,7 | 3,0 | 5,5 |
| TZISTR1161 | 13,5 | 87,6 | 87,6 | 0,2 | 220,4 | 14,8 | 46,1 | 14,6 | 33,9 | 26,5 | 2,3 | 14,4 | 0,1 | 82,3 | 3,3 | 5,3 |
| TZSTRI112 | 13,9 | 89,2 | 89,4 | 0,3 | 218,8 | 13,8 | 43,3 | 13,6 | 29,1 | 22,5 | 2,7 | 13,1 | 0,1 | 82,3 | 3,5 | 5,1 |
| CML548 | 13,5 | 83,5 | 84,7 | 1,0 | 219,1 | 16,1 | 42,5 | 13,1 | 36,0 | 21,8 | 3,3 | 13,9 | 0,1 | 82,0 | 3,8 | 5,0 |
| TZISTR1231 | 12,4 | 93,3 | 93,0 | -0,1 | 232,1 | 13,4 | 42,6 | 14,2 | 29,9 | 19,8 | 2,2 | 14,9 | 0,1 | 83,2 | 4,3 | 4,9 |
| 18 UK1-16 | 12,4 | 89,6 | 89,8 | 0,3 | 222,0 | 14,3 | 41,4 | 13,8 | 31,1 | 17,0 | 2,2 | 13,9 | 0,1 | 81,3 | 4,3 | 4,8 |
| CLHP0221 | 13,7 | 88,5 | 88,1 | -0,1 | 214,9 | 14,6 | 43,6 | 15,4 | 33,3 | 27,0 | 3,2 | 14,6 | 0,1 | 83,0 | 4,3 | 4,8 |
| TZISTR1275 | 11,6 | 98,0 | 98,7 | 0,4 | 273,8 | 14,2 | 39,5 | 12,3 | 31,1 | 22,0 | 2,6 | 16,9 | 0,1 | 80,3 | 3,5 | 4,7 |
| CLHP0156 | 12,7 | 87,6 | 87,8 | 0,3 | 223,7 | 15,5 | 43,0 | 12,7 | 34,8 | 19,5 | 2,5 | 13,5 | 0,1 | 79,8 | 3,5 | 4,6 |
| CLHP0113 | 13,9 | 90,3 | 90,0 | -0,1 | 196,9 | 13,6 | 43,7 | 15,6 | 32,9 | 17,5 | 2,3 | 14,3 | 0,1 | 79,5 | 3,5 | 4,5 |
| CML486 | 12,0 | 91,8 | 91,5 | -0,1 | 186,6 | 13,5 | 42,1 | 12,8 | 27,3 | 21,3 | 2,6 | 14,6 | 0,1 | 82,0 | 3,5 | 4,4 |
| 18 UK1-24 | 12,4 | 92,9 | 93,4 | 0,4 | 237,8 | 14,0 | 42,6 | 13,4 | 33,4 | 19,5 | 2,4 | 15,1 | 0,1 | 76,6 | 3,5 | 4,3 |
| CML541 | 13,1 | 89,4 | 89,3 | 0,0 | 195,5 | 12,8 | 39,4 | 15,0 | 30,5 | 22,8 | 2,1 | 14,0 | 0,1 | 80,4 | 4,3 | 4,1 |
| TZISTR1001 | 12,6 | 91,4 | 91,3 | 0,0 | 177,5 | 13,8 | 41,0 | 13,2 | 28,6 | 18,5 | 1,9 | 13,8 | 0,1 | 80,9 | 4,0 | 4,1 |
| CML390 | 11,8 | 84,6 | 84,7 | 0,3 | 173,4 | 13,2 | 41,7 | 12,3 | 26,8 | 13,3 | 1,8 | 14,9 | 0,1 | 81,2 | 4,3 | 3,9 |
| TZISTR1011 | 13,3 | 92,5 | 92,3 | -0,1 | 180,9 | 14,3 | 37,7 | 12,8 | 31,1 | 18,8 | 1,8 | 14,2 | 0,1 | 83,5 | 4,3 | 3,9 |
| TZISTR1157 | 9,2 | 85,7 | 85,5 | 0,0 | 211,0 | 14,5 | 43,2 | 14,2 | 32,7 | 16,3 | 1,9 | 13,5 | 0,1 | 82,7 | 3,8 | 3,8 |
| CML304 | 13,7 | 93,1 | 92,8 | -0,1 | 182,9 | 12,5 | 38,8 | 12,5 | 23,9 | 20,3 | 2,1 | 15,7 | 0,1 | 81,0 | 4,3 | 3,7 |
| 18 UK1-55 | 12,9 | 97,5 | 97,3 | -0,1 | 200,7 | 11,7 | 40,2 | 12,1 | 21,6 | 21,5 | 2,0 | 14,5 | 0,1 | 78,6 | 3,3 | 3,7 |
| 18 UK1-34 | 14,4 | 90,0 | 89,6 | -0,1 | 174,5 | 10,1 | 40,2 | 12,9 | 22,1 | 21,0 | 1,7 | 14,7 | 0,1 | 80,6 | 4,5 | 3,6 |
| 18 UK1-49 | 10,7 | 87,8 | 87,4 | -0,1 | 167,8 | 13,8 | 39,2 | 13,1 | 37,6 | 17,5 | 1,7 | 13,4 | 0,1 | 82,0 | 4,0 | 3,6 |
| TZISTR1160 | 13,9 | 91,2 | 91,7 | 0,4 | 178,6 | 13,7 | 37,3 | 11,8 | 21,3 | 21,5 | 1,6 | 14,1 | 0,1 | 75,2 | 3,8 | 3,5 |
| CML443 | 11,8 | 86,7 | 86,6 | 0,2 | 191,0 | 11,2 | 46,8 | 13,6 | 23,6 | 20,8 | 1,9 | 13,7 | 0,1 | 81,0 | 3,5 | 3,4 |
| TZISTR1004 | 13,5 | 89,0 | 89,4 | 0,4 | 232,1 | 13,6 | 44,0 | 13,4 | 28,4 | 20,3 | 1,8 | 14,3 | 0,1 | 82,8 | 4,0 | 3,4 |
| CLHP0058 | 10,3 | 89,6 | 89,4 | 0,0 | 182,1 | 12,2 | 37,2 | 13,8 | 25,9 | 17,0 | 1,6 | 13,7 | 0,1 | 78,4 | 4,0 | 3,4 |
| 18 UK1-1 | 10,3 | 91,6 | 91,5 | 0,0 | 172,4 | 11,3 | 39,2 | 12,6 | 25,8 | 15,5 | 1,5 | 13,3 | 0,1 | 78,9 | 4,5 | 3,4 |
| TZISTR1263 | 12,4 | 90,9 | 90,6 | -0,1 | 210,0 | 12,8 | 41,0 | 13,5 | 27,8 | 16,3 | 1,6 | 14,5 | 0,1 | 78,8 | 4,0 | 3,4 |
| TZISTR1165 | 12,0 | 91,4 | 93,4 | 1,3 | 218,6 | 15,7 | 44,0 | 12,3 | 29,9 | 12,8 | 1,7 | 15,5 | 0,1 | 78,5 | 4,3 | 3,3 |
| 18 UK1-8 | 8,4 | 91,8 | 91,7 | 0,0 | 223,6 | 12,5 | 39,8 | 14,0 | 29,3 | 12,0 | 1,7 | 14,3 | 0,1 | 79,7 | 4,5 | 3,3 |
| 18 UK1-29 | 11,8 | 90,3 | 91,1 | 0,6 | 213,7 | 15,2 | 41,6 | 13,2 | 26,6 | 14,8 | 1,5 | 14,3 | 0,1 | 77,2 | 4,0 | 3,3 |
| TZISTR1199 | 12,0 | 89,2 | 89,6 | 0,4 | 163,0 | 11,8 | 42,7 | 13,5 | 21,9 | 11,8 | 1,7 | 14,7 | 0,1 | 78,1 | 5,0 | 3,3 |
| I-137 | 11,0 | 89,4 | 89,4 | 0,2 | 163,4 | 11,2 | 43,7 | 14,5 | 24,7 | 13,0 | 1,5 | 13,6 | 0,1 | 77,2 | 4,5 | 3,3 |
| CLHP0022 | 11,8 | 90,3 | 90,9 | 0,5 | 160,2 | 11,5 | 34,7 | 11,4 | 20,6 | 16,3 | 1,5 | 15,2 | 0,1 | 74,3 | 4,5 | 3,2 |
| 18 UK1-2-12 | 11,6 | 83,0 | 84,4 | 1,1 | 164,6 | 10,1 | 35,3 | 12,0 | 24,1 | 17,5 | 1,4 | 12,8 | 0,1 | 82,4 | 5,3 | 3,2 |
| 18 UK1-56 | 12,2 | 90,7 | 90,6 | 0,0 | 187,2 | 11,3 | 37,3 | 11,9 | 24,8 | 23,0 | 1,5 | 13,2 | 0,1 | 82,3 | 4,3 | 3,2 |
| CML544 | 11,0 | 89,8 | 89,6 | 0,0 | 190,9 | 11,4 | 40,6 | 13,0 | 24,7 | 16,8 | 1,5 | 13,5 | 0,1 | 77,6 | 3,8 | 3,2 |
| CLHP0343 | 10,9 | 84,3 | 84,4 | 0,3 | 169,0 | 11,7 | 38,5 | 13,1 | 27,4 | 21,8 | 1,6 | 12,9 | 0,1 | 83,4 | 4,5 | 3,2 |
| CML539 | 13,3 | 85,4 | 86,2 | 0,7 | 216,2 | 12,9 | 40,4 | 12,4 | 24,1 | 16,0 | 1,4 | 13,2 | 0,1 | 79,9 | 4,5 | 3,1 |
| 18 UK1-57 | 9,3 | 91,8 | 91,9 | 0,2 | 197,2 | 9,8 | 40,4 | 13,2 | 21,2 | 10,8 | 1,5 | 14,2 | 0,1 | 78,9 | 4,0 | 3,1 |
| 18 UK1-3 | 9,9 | 96,6 | 99,0 | 1,6 | 190,3 | 10,9 | 38,1 | 11,8 | 19,8 | 10,5 | 1,5 | 15,8 | 0,1 | 76,2 | 3,8 | 3,1 |
| CLHP0312 | 9,3 | 93,6 | 94,1 | 0,4 | 138,6 | 11,4 | 42,4 | 13,7 | 27,1 | 13,8 | 1,4 | 14,5 | 0,1 | 81,7 | 4,3 | 3,0 |
| 18 UK1-15 | 13,3 | 91,4 | 91,9 | 0,4 | 161,0 | 9,7 | 38,5 | 13,4 | 21,4 | 22,5 | 1,4 | 13,6 | 0,1 | 80,8 | 4,8 | 3,0 |
| CLHP0020 | 10,5 | 95,5 | 96,8 | 0,9 | 162,9 | 11,4 | 43,5 | 16,1 | 24,4 | 13,5 | 1,4 | 13,6 | 0,1 | 77,1 | 4,0 | 2,9 |
| 18 UK1-42 | 13,5 | 90,3 | 90,6 | 0,3 | 167,3 | 9,8 | 39,7 | 12,5 | 21,2 | 18,3 | 1,4 | 13,6 | 0,1 | 79,1 | 3,8 | 2,9 |
| TZSTRI111 | 10,7 | 92,3 | 92,3 | 0,0 | 182,5 | 9,7 | 42,0 | 17,0 | 22,1 | 16,3 | 1,7 | 15,2 | 0,1 | 80,0 | 4,0 | 2,9 |
| TZISTR1163 | 10,3 | 90,3 | 90,4 | 0,2 | 165,2 | 9,6 | 37,8 | 12,4 | 19,8 | 13,8 | 1,3 | 13,2 | 0,1 | 75,1 | 4,0 | 2,9 |
| TZSTRI110 | 12,4 | 89,0 | 89,6 | 0,5 | 182,0 | 10,6 | 40,2 | 12,5 | 22,4 | 15,0 | 1,4 | 14,5 | 0,1 | 79,7 | 4,8 | 2,9 |
| CML537 | 11,0 | 92,7 | 92,5 | -0,1 | 179,6 | 11,0 | 41,6 | 14,2 | 23,8 | 12,5 | 1,2 | 14,1 | 0,1 | 82,5 | 4,3 | 2,8 |
| TZISTR1164 | 12,9 | 90,3 | 90,6 | 0,3 | 168,1 | 10,7 | 40,2 | 14,6 | 20,3 | 17,0 | 1,3 | 13,5 | 0,1 | 75,9 | 4,5 | 2,8 |
| HA04A-2107-36 | 11,6 | 93,4 | 94,0 | 0,4 | 181,3 | 12,7 | 43,1 | 13,9 | 24,4 | 15,0 | 1,3 | 15,2 | 0,1 | 78,5 | 4,0 | 2,8 |
| TZISTR25 | 9,9 | 87,9 | 87,9 | 0,2 | 180,3 | 12,7 | 40,7 | 13,8 | 24,2 | 13,8 | 1,5 | 13,7 | 0,1 | 79,3 | 4,3 | 2,8 |
| CML312 | 12,0 | 85,0 | 86,1 | 0,9 | 183,4 | 12,1 | 38,5 | 13,5 | 27,7 | 15,0 | 1,3 | 13,1 | 0,1 | 80,7 | 4,8 | 2,7 |
| TZISTR1262 | 10,5 | 92,5 | 92,6 | 0,2 | 180,9 | 10,6 | 41,3 | 14,1 | 22,3 | 12,0 | 1,3 | 13,4 | 0,1 | 81,3 | 5,0 | 2,7 |
| TZDEEI50 | 11,2 | 87,4 | 87,0 | -0,1 | 168,3 | 10,5 | 40,3 | 17,4 | 22,3 | 16,0 | 1,3 | 15,3 | 0,1 | 74,7 | 5,0 | 2,7 |
| CLHP0303 | 12,2 | 96,2 | 96,2 | 0,0 | 180,8 | 12,4 | 39,2 | 13,1 | 22,5 | 12,0 | 1,3 | 15,5 | 0,1 | 74,7 | 4,0 | 2,7 |
| MAK1-122 | 10,9 | 99,5 | 101,1 | 1,0 | 223,9 | 12,4 | 36,6 | 10,7 | 19,3 | 10,8 | 1,3 | 15,3 | 0,1 | 70,2 | 3,8 | 2,7 |
| TZISTR1159 | 12,6 | 90,5 | 90,4 | 0,0 | 166,0 | 16,0 | 40,9 | 12,2 | 27,6 | 15,0 | 1,4 | 14,7 | 0,1 | 77,5 | 4,3 | 2,7 |
| TZSTRI113 | 11,4 | 89,8 | 90,2 | 0,4 | 201,6 | 13,8 | 36,1 | 10,8 | 26,4 | 14,5 | 1,1 | 14,0 | 0,1 | 80,8 | 5,0 | 2,6 |
| CML441 | 11,8 | 92,9 | 93,2 | 0,3 | 158,5 | 9,8 | 37,2 | 14,9 | 21,1 | 15,8 | 1,1 | 14,4 | 0,1 | 80,0 | 4,8 | 2,6 |
| TZEEI34 | 12,6 | 89,6 | 89,6 | 0,2 | 188,2 | 13,1 | 39,6 | 13,8 | 29,5 | 20,3 | 1,3 | 14,5 | 0,1 | 78,7 | 3,8 | 2,6 |
| CML550 | 11,0 | 92,3 | 92,5 | 0,2 | 185,4 | 11,5 | 38,8 | 12,9 | 24,7 | 13,3 | 1,2 | 14,2 | 0,1 | 81,7 | 4,5 | 2,6 |
| TZSTRI115 | 11,4 | 94,0 | 95,3 | 0,9 | 237,0 | 11,7 | 39,1 | 11,8 | 21,7 | 18,0 | 1,5 | 15,5 | 0,0 | 73,6 | 3,8 | 2,5 |
| TZISTR1003 | 9,7 | 94,7 | 95,7 | 0,6 | 184,3 | 12,0 | 37,1 | 13,6 | 21,7 | 13,8 | 1,2 | 15,6 | 0,1 | 79,6 | 4,0 | 2,5 |
| CLHP0003 | 12,4 | 86,5 | 85,7 | -0,3 | 161,4 | 11,6 | 43,6 | 15,8 | 22,9 | 14,8 | 1,2 | 13,7 | 0,1 | 76,8 | 4,5 | 2,5 |
| CLHP0310 | 12,6 | 93,4 | 94,1 | 0,5 | 218,4 | 11,7 | 40,3 | 13,4 | 23,4 | 14,0 | 1,2 | 14,8 | 0,1 | 77,6 | 4,3 | 2,5 |
| 18 UK1-46 | 10,1 | 90,5 | 91,7 | 0,9 | 144,2 | 9,6 | 36,9 | 12,7 | 17,4 | 15,5 | 1,1 | 13,1 | 0,0 | 72,1 | 3,8 | 2,5 |
| 18 UK1-2-13 | 13,3 | 96,4 | 96,6 | 0,2 | 156,7 | 12,0 | 37,8 | 13,8 | 22,4 | 12,0 | 1,2 | 15,8 | 0,1 | 76,9 | 4,8 | 2,5 |
| TZISTR1232 | 11,0 | 94,5 | 94,3 | -0,1 | 178,0 | 10,3 | 38,5 | 13,1 | 23,3 | 12,8 | 1,1 | 14,3 | 0,1 | 79,5 | 5,0 | 2,5 |
| TZISTR1177 | 11,4 | 90,3 | 90,4 | 0,2 | 131,7 | 10,1 | 36,2 | 12,9 | 22,2 | 16,0 | 1,1 | 13,1 | 0,0 | 78,9 | 4,8 | 2,4 |
| TZSTRI108 | 12,4 | 91,4 | 91,9 | 0,4 | 180,2 | 13,0 | 37,1 | 11,8 | 20,2 | 15,0 | 1,1 | 13,9 | 0,1 | 82,1 | 4,8 | 2,4 |
| CLHP0302 | 11,2 | 88,9 | 88,7 | 0,0 | 152,5 | 11,7 | 36,4 | 11,5 | 18,3 | 15,5 | 1,1 | 13,4 | 0,1 | 78,0 | 5,3 | 2,4 |
| 18 UK1-14 | 12,2 | 89,8 | 89,4 | -0,1 | 148,1 | 9,9 | 38,8 | 13,6 | 21,9 | 14,5 | 1,1 | 13,2 | 0,1 | 78,6 | 5,0 | 2,4 |
| CML451 | 8,4 | 89,0 | 89,1 | 0,2 | 163,0 | 11,8 | 33,4 | 12,1 | 26,1 | 12,5 | 1,1 | 13,3 | 0,1 | 82,6 | 4,3 | 2,4 |
| TZISTR1174 | 9,0 | 88,5 | 88,7 | 0,3 | 177,2 | 13,7 | 38,1 | 13,1 | 27,5 | 11,0 | 1,1 | 14,1 | 0,1 | 79,4 | 5,3 | 2,4 |
| 18 UK1-17 | 11,2 | 91,1 | 90,9 | 0,0 | 173,0 | 11,3 | 37,7 | 12,7 | 21,8 | 14,8 | 1,1 | 13,4 | 0,1 | 79,1 | 5,0 | 2,4 |
| 18 UK1-5 | 10,3 | 93,6 | 93,6 | 0,0 | 161,5 | 10,2 | 37,5 | 11,8 | 20,3 | 13,3 | 1,1 | 13,9 | 0,0 | 76,5 | 5,3 | 2,4 |
| CML538 | 12,0 | 92,7 | 93,8 | 0,7 | 178,4 | 12,0 | 39,2 | 13,3 | 22,3 | 13,0 | 1,1 | 14,7 | 0,1 | 78,3 | 4,3 | 2,3 |
| TZSTRI102 | 12,9 | 85,0 | 87,6 | 1,8 | 160,2 | 12,1 | 41,4 | 12,5 | 26,4 | 15,0 | 1,1 | 12,9 | 0,1 | 81,5 | 4,8 | 2,3 |
| 18 UK1-9 | 11,8 | 85,9 | 87,9 | 1,4 | 187,1 | 13,7 | 38,9 | 12,8 | 26,5 | 12,0 | 1,1 | 12,8 | 0,1 | 81,2 | 4,5 | 2,3 |
| 17CED MAK1-61/62 | 10,3 | 94,0 | 96,4 | 1,6 | 171,2 | 12,9 | 39,3 | 13,2 | 26,9 | 13,0 | 1,1 | 15,2 | 0,1 | 77,5 | 4,5 | 2,3 |
| TZISTR1248 | 12,2 | 94,9 | 94,7 | -0,1 | 210,4 | 10,1 | 40,3 | 14,1 | 22,4 | 17,3 | 1,1 | 13,7 | 0,0 | 76,9 | 4,3 | 2,3 |
| 18 UK1-37 | 9,9 | 89,6 | 89,3 | -0,1 | 170,6 | 9,6 | 33,7 | 11,7 | 19,1 | 16,3 | 1,0 | 14,4 | 0,1 | 80,5 | 5,5 | 2,3 |
| 18 UK1-18 | 10,3 | 96,0 | 96,4 | 0,3 | 201,9 | 9,8 | 36,6 | 13,4 | 20,5 | 16,0 | 1,1 | 14,2 | 0,0 | 79,8 | 3,8 | 2,3 |
| CLHP0005 | 11,8 | 93,1 | 92,3 | -0,4 | 186,5 | 11,1 | 36,2 | 11,9 | 19,7 | 16,0 | 1,0 | 13,9 | 0,1 | 83,3 | 4,5 | 2,3 |
| CML566 | 12,0 | 90,9 | 90,9 | 0,2 | 177,3 | 10,9 | 36,3 | 12,9 | 24,1 | 18,3 | 1,0 | 12,9 | 0,0 | 83,1 | 4,3 | 2,2 |
| CLHP00378 | 11,4 | 90,5 | 90,8 | 0,3 | 150,9 | 10,9 | 39,6 | 13,4 | 22,2 | 12,5 | 1,0 | 13,3 | 0,1 | 78,3 | 5,0 | 2,2 |
| CML536 | 9,7 | 90,1 | 90,6 | 0,4 | 154,2 | 10,1 | 41,9 | 13,0 | 21,1 | 9,8 | 0,9 | 13,2 | 0,0 | 74,0 | 5,3 | 2,2 |
| TZISTR1175 | 12,2 | 94,4 | 96,2 | 1,2 | 161,9 | 10,8 | 39,7 | 13,3 | 23,8 | 14,8 | 0,9 | 14,8 | 0,1 | 79,9 | 4,5 | 2,2 |
| TZSTRI114 | 12,6 | 92,0 | 92,3 | 0,3 | 163,6 | 11,6 | 41,6 | 13,5 | 22,1 | 15,0 | 1,0 | 13,3 | 0,1 | 78,7 | 4,5 | 2,2 |
| CML547 | 10,9 | 91,8 | 93,2 | 1,0 | 154,7 | 9,8 | 39,9 | 13,5 | 23,1 | 12,0 | 1,0 | 13,3 | 0,1 | 78,1 | 4,0 | 2,2 |
| TZISTR1166 | 10,9 | 94,9 | 94,9 | 0,0 | 146,6 | 11,1 | 37,9 | 12,6 | 18,0 | 13,5 | 0,7 | 13,7 | 0,0 | 74,2 | 4,5 | 2,1 |
| TZEEI21 | 9,5 | 92,9 | 92,6 | -0,1 | 157,2 | 10,0 | 38,1 | 16,0 | 20,6 | 10,8 | 1,0 | 15,9 | 0,0 | 76,5 | 4,8 | 2,1 |
| TZSTRI109 | 9,0 | 93,6 | 93,4 | -0,1 | 174,2 | 9,6 | 34,2 | 11,5 | 19,3 | 10,5 | 1,0 | 15,1 | 0,0 | 82,9 | 4,5 | 2,0 |
| 18 UK1-21 | 7,5 | 92,9 | 92,6 | -0,1 | 165,9 | 9,5 | 32,4 | 11,8 | 20,7 | 10,0 | 0,9 | 13,9 | 0,0 | 82,5 | 4,3 | 2,0 |
| TZISTR1018 | 11,2 | 89,8 | 89,6 | 0,0 | 158,6 | 10,3 | 35,6 | 12,5 | 21,5 | 11,3 | 0,9 | 13,1 | 0,1 | 82,8 | 5,5 | 2,0 |
| TZEEI14 | 10,3 | 93,3 | 93,4 | 0,2 | 151,4 | 9,1 | 35,9 | 12,4 | 18,9 | 15,0 | 0,9 | 13,8 | 0,0 | 79,3 | 5,5 | 2,0 |
| CML442 | 12,0 | 96,9 | 98,7 | 1,1 | 174,0 | 13,4 | 37,2 | 12,7 | 23,2 | 17,0 | 0,9 | 13,8 | 0,0 | 68,8 | 4,5 | 2,0 |
| CLHP0049 | 8,2 | 85,2 | 86,2 | 0,9 | 144,9 | 7,5 | 24,3 | 7,5 | 17,1 | 7,5 | 0,9 | 13,2 | 0,0 | 79,2 | 5,0 | 2,0 |
| 18 UK3-2-2 | 7,1 | 89,8 | 89,8 | 0,2 | 143,9 | 9,1 | 38,9 | 12,2 | 20,5 | 9,8 | 0,9 | 13,2 | 0,1 | 79,7 | 4,8 | 2,0 |
| 18 UK1-33 | 8,4 | 92,7 | 93,0 | 0,3 | 179,4 | 10,5 | 43,8 | 12,3 | 22,5 | 13,3 | 0,9 | 13,7 | 0,0 | 79,3 | 6,0 | 1,9 |
| CLHP0326 | 4,6 | 90,0 | 90,8 | 0,6 | 152,3 | 6,3 | 21,7 | 6,2 | 11,5 | 3,3 | 0,9 | 14,7 | 0,0 | 78,0 | 5,3 | 1,9 |
| 18 UK1-35 | 9,7 | 92,0 | 92,5 | 0,4 | 144,4 | 9,0 | 38,1 | 15,0 | 21,0 | 12,3 | 0,9 | 13,4 | 0,0 | 76,1 | 5,0 | 1,9 |
| CML504 | 7,6 | 88,5 | 90,0 | 1,1 | 142,4 | 9,5 | 33,6 | 8,5 | 19,2 | 7,3 | 0,8 | 13,0 | 0,0 | 76,7 | 4,5 | 1,9 |
| 18 UK1-32 | 9,3 | 89,6 | 89,4 | 0,0 | 128,9 | 8,7 | 30,4 | 10,2 | 15,7 | 10,5 | 0,8 | 12,9 | 0,0 | 73,6 | 5,0 | 1,9 |
| 18 UK1-2-10 | 8,2 | 91,8 | 92,1 | 0,3 | 182,2 | 10,8 | 38,0 | 14,4 | 27,3 | 8,8 | 0,9 | 13,6 | 0,1 | 84,4 | 5,0 | 1,9 |
| CLHP0364 | 9,3 | 86,8 | 87,0 | 0,3 | 157,9 | 9,6 | 32,6 | 10,9 | 19,3 | 10,8 | 0,9 | 12,7 | 0,0 | 78,9 | 4,8 | 1,9 |
| 17CED MAK1-59/60 | 11,6 | 93,1 | 92,6 | -0,2 | 181,7 | 10,8 | 33,5 | 11,4 | 22,4 | 13,8 | 0,9 | 13,4 | 0,0 | 80,2 | 5,0 | 1,8 |
| CML542 | 11,2 | 88,5 | 89,6 | 0,9 | 175,0 | 11,3 | 38,1 | 13,3 | 25,1 | 10,5 | 0,8 | 12,8 | 0,1 | 80,9 | 5,3 | 1,8 |
| TZISTR1154 | 9,7 | 90,9 | 90,9 | 0,2 | 175,7 | 9,6 | 38,0 | 10,3 | 20,3 | 9,3 | 0,8 | 13,3 | 0,0 | 78,9 | 4,8 | 1,8 |
| TZISTR1244 | 8,8 | 91,4 | 91,1 | -0,1 | 188,9 | 9,5 | 39,0 | 15,3 | 21,0 | 12,8 | 0,8 | 13,1 | 0,0 | 78,5 | 4,5 | 1,8 |
| TZDEEI55 | 11,6 | 90,0 | 89,8 | 0,0 | 141,5 | 9,8 | 38,5 | 13,9 | 17,5 | 14,0 | 0,9 | 13,1 | 0,0 | 76,3 | 5,3 | 1,8 |
| 17CED MAK1-48/47 | 10,3 | 93,6 | 95,7 | 1,3 | 204,9 | 11,8 | 38,0 | 10,8 | 20,7 | 11,8 | 0,8 | 14,5 | 0,0 | 74,2 | 4,8 | 1,8 |
| A1220-4CYL | 11,4 | 94,4 | 94,7 | 0,3 | 169,6 | 12,4 | 36,0 | 12,6 | 21,9 | 11,5 | 1,0 | 14,0 | 0,0 | 78,5 | 4,3 | 1,7 |
| TZEEI10 | 13,5 | 85,0 | 85,5 | 0,5 | 174,2 | 11,0 | 36,5 | 12,8 | 19,6 | 14,3 | 0,8 | 13,7 | 0,1 | 77,8 | 4,8 | 1,6 |
| CLHP0350 | 9,3 | 87,2 | 87,4 | 0,3 | 169,3 | 10,1 | 31,0 | 11,7 | 21,0 | 9,5 | 0,8 | 12,5 | 0,0 | 81,1 | 5,3 | 1,6 |
| CML440 | 11,6 | 89,4 | 89,3 | 0,0 | 155,7 | 10,0 | 35,9 | 12,2 | 17,3 | 10,8 | 0,8 | 12,6 | 0,0 | 78,5 | 5,3 | 1,6 |
| TZISTR1224 | 11,8 | 91,4 | 91,9 | 0,4 | 145,3 | 9,4 | 31,7 | 11,7 | 21,6 | 14,5 | 0,8 | 13,2 | 0,0 | 71,4 | 5,3 | 1,6 |
| 18 UK1-6 | 8,2 | 90,7 | 91,5 | 0,6 | 141,2 | 10,6 | 29,5 | 10,6 | 17,8 | 6,8 | 0,7 | 13,0 | 0,0 | 72,4 | 5,5 | 1,4 |
| TZISTR1223 | 9,3 | 89,8 | 89,6 | 0,0 | 133,3 | 8,6 | 29,6 | 13,5 | 17,5 | 7,3 | 0,7 | 13,6 | 0,0 | 80,3 | 5,3 | 1,4 |
| TZSTRI117 | 8,8 | 82,8 | 83,8 | 0,9 | 169,8 | 11,7 | 36,4 | 12,1 | 17,1 | 10,0 | 0,6 | 12,3 | 0,0 | 72,5 | 5,3 | 1,4 |
| TZSTRI104 | 6,1 | 94,7 | 94,9 | 0,2 | 132,3 | 8,2 | 26,2 | 9,4 | 13,4 | 5,5 | 0,6 | 14,1 | 0,0 | 73,9 | 6,0 | 1,3 |
| TZISTR1162 | 6,9 | 93,4 | 93,6 | 0,2 | 145,1 | 8,2 | 27,6 | 9,6 | 18,8 | 9,5 | 0,6 | 14,4 | 0,0 | 75,1 | 6,0 | 1,3 |
| 18 UK1-54 | 7,6 | 83,5 | 86,1 | 1,8 | 154,1 | 7,0 | 22,8 | 8,0 | 14,5 | 6,0 | 0,4 | 12,6 | 0,0 | 82,2 | 5,8 | 0,9 |
| CML545 | 6,5 | 95,6 | 97,0 | 0,9 | 98,9 | 8,1 | 25,5 | 8,5 | 12,7 | 5,0 | 0,4 | 12,7 | 0,0 | 73,3 | 6,3 | 0,9 |

**Table S3: Clustering of 128 maize genotypes based on phenotypic traits**

| **Cluster** | **Entry** | **% Membership** |
| --- | --- | --- |
| I | 1 2 5 6 7 8 9 11 12 13 14 15 17 18 19 21 23 24 25 27 28 29 31 32 34 35 36 37 38 39 40 41 42 45 46 47 48 50 52 53 54 55 56 57 58 60 61 63 64 65 66 68 70 71 72 74 75 76 77 78 79 80 81 82 83 84 85 86 87 88 89 90 91 92 93 94 95 96 97 99 100 101 102 103 104 105 107 108 109 110 111 112 116 117 118 119 121 122 124 125 126 127 128 | 81% |
| II | 3 10 20 26 67 69 73 106 113 114 120 123 | 9% |
| III | 4 16 22 30 33 43 44 49 51 59 62 98 115 | 10% |

**Table S4. Clustering of 128 maize genotypes based on 11405 SNP markers**

| **Cluster** | **Entry** | **% Membership** |
| --- | --- | --- |
| I | 1 64 76 77 78 79 80 81 82 83 84 85 86 87 88 89 90 91 92 93 94 95 96 97 98 99 100 101 127 128 | 23 % |
| II | 2 12 15 17 20 21 23 25 27 29 31 36 40 41 43 49 52 56 58 60 61 63 68 69 70 74 105 108 109 110 118 121 123 | 26% |
| III | 3 4 5 6 7 8 9 10 11 13 14 16 18 19 22 24 26 28 30 32 33 34 35 37 38 39 42 44 45 46 47 48 50 51 53 54 55 57 59 62 65 66 67 71 72 73 75 102 103 104 106 107 111 112 113 114 115 116 117 119 120 122 124 125 126 | 51% |
